# Impacts of genetic selection on satellite cell function in poultry

**DOI:** 10.3389/fphys.2026.1807624

**Published:** 2026-04-22

**Authors:** Joseph Yimiletey, Sandra G. Velleman, Hui Yu

**Affiliations:** Department of Animal Sciences, The Ohio State University, Columbus, OH, United States

**Keywords:** genetic selection, heterogeneity, proliferation, satellite cell, transcriptome, wooden breast

## Abstract

Over the past several decades, intensive genetic selection has markedly increased growth rate, breast muscle yield, and production efficiency in commercial broilers. These gains have been accompanied by substantial changes in muscle organization, physiology, metabolism, and cellular regulation. Satellite cells, the resident muscle stem cells, play a key role in posthatch muscle growth by supplying nuclei to adjacent growing myofibers, thereby supporting the muscle hypertrophy process. This review summarizes the role of satellite cells in poultry muscle development and discusses how genetic selection for rapid muscle growth has reshaped satellite cell proliferation, differentiation, molecular profiles, and functional heterogeneity, with important consequences for muscle growth and quality. Moreover, increasing evidence suggests that changes in satellite cell function associated with genetic selection may contribute to the development of breast muscle myopathies, particularly wooden breast in broiler chickens. Moving forward, continued refinement of poultry production systems will benefit from deeper insights into satellite cell biology to support efficient muscle growth while reducing the occurrence of muscle myopathies.

## Introduction

The domestic chicken (*Gallus gallus domesticus*) traces its ancestry to both the red and gray junglefowl, with domestication believed to have occurred between 5,000 and 10,000 years ago (Eriksson et al., 2008; Siegel, 2014). Selective breeding of chickens has been practiced since at least 1873, marked by the establishment of the American Poultry Association (Ekarius, 2007). A major turning point in poultry production occurred in 1945 with the launch of the “Chicken of Tomorrow” competition, which marked the beginning of the modern broiler industry. Winning lines from this event were subsequently crossbred to produce the first commercial broiler hybrids (Loy, 1952). Since then, intensive genetic selection has been directed toward developing broilers with rapid growth, higher breast muscle yield, and improved feed efficiency, leading to substantial improvements in production traits (Havenstein et al., 2003a; 2003b; Zuidhof et al., 2014). Studies have shown that the time required for broilers to reach market weight has decreased substantially, from approximately 10–12 weeks in the 1950s to 6–7 weeks by the 1990s, and further to about 5–6 weeks in modern production systems (Schmidt et al., 2009; Concil, 2022). In parallel, genetic selection has markedly increased breast muscle (p.major) proportion, rising from approximately 9% of total body mass at 5 weeks of age in a heritage line unselected since the 1950s to nearly 18% in modern Ross 708 broilers (Schmidt et al., 2009). This dramatic increase in muscle mass is achieved largely through post-hatch hypertrophic growth, which depends on satellite cells (**SCs**) that donate nuclei to existing muscle fibers, thereby supporting enhanced transcriptional and protein synthesis capacity (Campion, 1984; Schultz and McCormick, 1994; Halevy et al., 2004). This review explores how genetic selection alters SC function, regulation, and heterogeneity, and how these changes ultimately influence muscle growth and quality. The discussion focuses primarily on broilers, with extension to turkeys given the application of similar selection strategies.

## Role of satellite cells in muscle growth in poultry

Satellite cells were first identified by Mauro in (1961) and described as being “wedged” between the basement membrane and the plasma membrane of muscle fibers. In chickens, SCs are present in low numbers at embryonic day 14 but increase markedly during late embryogenesis and the early post-hatch period. Their activity peaks around hatching, after which their proliferative capacity declines rapidly (Hartley et al., 1992; Halevy et al., 2000). During this critical development window, SCs are highly sensitive to temperature fluctuations with both cold and heat reduce their proliferation and differentiation rate (Xu et al., 2021a), and can also shift their cellular fate decisions (Xu et al., 2022a). By two weeks of age, SCs become largely quiescent and are reactivated only in response to stimuli that promote muscle growth or muscle repair (Mozdziak et al., 2002; Clark et al., 2017). In avian species, the number of muscle fibers is fixed at hatch; therefore, post-hatch muscle growth is driven mainly by hypertrophy (increases in fiber size), during which SCs contribute nuclei to adjacent muscle fibers, increasing the genetic capacity needed to support hypertrophic growth (White et al., 2010; Rehfeldt et al., 2011).

Sex differences have a significant impact on SC biology and muscle growth in poultry. Male broilers are generally heavier than females, whereas females often exhibit a higher proportion of breast muscle yield. Consistent with these differences, a previous study reported that at post-hatch day 56, males have a greater number of breast muscle fibers, but a smaller fiber diameter compared with females (Orlowski et al., 2021). Transcriptomic analysis of p. major muscles from male and female broilers at three weeks of age, a stage when differences in body weight and growth rate are not yet apparent, identified 260 genes differentially expressed between sexes (Brothers et al., 2019). Beyond broilers, sexual dimorphism in muscle development is also evident in turkeys. A previous study comparing SCs isolated from the F-line (selected for increased 16-week body weight) and a randombred control turkey line found that males from the control line exhibited significantly higher proliferation rates than females. In contrast, F-line males showed greater differentiation capacity, as indicated by the formation of significantly longer myotubes (Velleman et al., 2000). A follow-up study further demonstrated that, in both lines, females exhibited significantly greater proportions of Syndecan4-positive and Glypican1-positive SCs during differentiation, whereas males exhibited significantly greater proportions of these SC populations during proliferation (Song et al., 2013).

## Genetic selection alters satellite cell proliferation and differentiation

Satellite cell proliferation and differentiation are essential because they control the supply of myogenic cells and nuclei required for post-hatch muscle hypertrophy and regeneration, ultimately influencing both growth performance and muscle quality. Several lines of evidence have been shown that genetic selection has greatly altered the SC proliferation and differentiation capabilities. Xu and Velleman (2023) compared SC proliferation and differentiation in cells isolated from the p. major muscle of a modern commercial broiler line (MC), a Cornish Rock line (BPM8), and a randombred line (RBch) in the 1990s. Satellite cells from the MC line showed significantly reduced proliferation at 72 h and consistently lower differentiation across all examined time points (0–72 h) compared with BPM8 and RBch. These functional differences were accompanied by decreased expression of key myogenic regulators, including *myoblast determination factor 1* (*MyoD*) and *myogenin* (*MyoG*), as well as lower *mTOR* mRNA levels in MC line SCs, suggesting reduced hypertrophic and regenerative potential. Knockdown of mTOR or Fzd7 impaired SC activity across lines, although MC line SCs were generally less responsive, and proliferation in the MC line appeared more dependent on Fzd7-mediated Wnt/PCP signaling than differentiation (Xu and Velleman, 2023).

Consistent with these observations, a long-term selection model in White Plymouth Rock chickens based on 56-day body weight (high-weight selected [HWS] vs. low-weight selected [LWS]) similarly demonstrated that selection for increased growth profoundly alters SC biology. Specifically, SCs isolated from the p. major of HWS birds at 5 and 8 weeks of age displayed impaired proliferative and differentiative capacity compared with SCs from the LWS line (Daughtry et al., 2017). Interestingly, SCs isolated from 5-day-old chicks from the same lines showed the opposite pattern, indicating that the effects of genetic selection on SC function vary across developmental stages and with the progression of muscle hypertrophy (Geiger et al., 2018).

In contrast to broilers, although similar genetic selection strategies have been applied in turkeys, these approaches appear to influence turkey SC biology differently. Studies compared SCs isolated from the p. major muscle of a turkey line selected for increased 16-week body weight (F-line) and a random-bred slow-growing control line (RBC2) suggested that F-line cells proliferated at a faster rate than the RBC2 line (Velleman et al., 2000). Similar trends have been reported when comparing RBC2 with the Nicholas commercial turkey (NCT) line. Specifically, SCs from NCT turkeys exhibit significantly greater proliferation and myogenic differentiation, along with increased myotube diameter and lipid accumulation, relative to the non-selected RBC2 line (Xu et al., 2021a; 2022b). Nevertheless, the factors driving these divergent SC responses to genetic selection between boilers and turkeys remain to be determined.

Satellite cells possess inherent plasticity and, in response to specific nutritional and environmental stimuli, can differentiate into mesenchymal lineages other than the myogenic lineage, including adipocytes (Asakura et al., 2001). In broiler chicks, feed restriction during the first week posthatch, a critical window of satellite cell proliferation, induces marked lipid synthesis and fat accumulation, contributing to intramuscular fat deposition in the breast muscle (Velleman et al., 2014). This response is considered detrimental because it reduces the protein-to-fat ratio of poultry breast meat. Similarly, previous studies have reported that heat stress can shift SC fate toward adipogenic differentiation, with lipid content being greater in SC from the NCT line than in those from the RBC2 line at 43 °C (Xu et al., 2021b). A proposed mechanism underlying the effects of genetic selection on SC biology and function is the associated increase in muscle fiber size. The development of giant fibers may reduce connective tissue area and capillary density, thereby restricting oxygen and nutrient delivery and limiting the clearance of metabolic waste products such as lactic acid. Together, these changes may promote muscle damage and further impair normal SC function (Wilson et al., 1990; Velleman and Nestor, 2003; Velleman and Coy, 2020).

## Genetic selection alters satellite cell transcriptome

Genetic selection has significantly altered the p. major transcriptome. In comparison of modern and legacy broilers at post-hatch days 6 and 21, 189 genes were differentially expressed at day 6 and 193 genes at day 21. At day 6, *insulin like growth factor 1* (*IGF1*) and its receptor (*IGF1R*) were enriched in modern broilers, whereas *myostatin* (*MSTN*) and *angiotensin I converting enzyme 1* (*ACE*) were enriched in legacy birds. By day 21, *IGF1R* and *MSTN* showed more similar expression between the two lines, but *IGF1* remained higher in modern broilers, while *ACE* remained higher in legacy broilers (Davis et al., 2015). Interestingly, elevated *ACE* expression was also reported in a separate study comparing an unselected foundation line (Barred Plymouth Rock) with modern pedigree males at an immature adult stage (≤8 weeks old), which identified more than 2,000 differentially expressed genes between the lines (Kong et al., 2017).

Zooming in on SCs, foundational work in turkeys has provided important insights into how genetic background shapes SC molecular programs, particularly under varying thermal conditions. Molecular profiling of SCs from RBC2 and NCT lines revealed pronounced divergence in gene expression. During differentiation, more than 250 genes were differentially expressed, with most transcripts downregulated in NCT cells (Reed et al., 2022b). Genes upregulated in NCT SCs were significantly enriched for GO biological processes related to mitochondrial energy production, including mitochondrial ATP synthesis coupled electron transport, ATP synthesis coupled electron transport, and the aerobic electron transport chain. Even larger differences were observed during proliferation, with over 600 genes differentially expressed between the lines (Reed et al., 2022a), and gene ontology analysis indicating enrichment of pathways involved in cell differentiation and morphogenesis in the RBC2 line. These line-dependent differences also extended beyond mRNA expression to circular RNA profiles (Powell et al., 2024), with 15 differentially expressed circular RNAs identified during proliferation (predicted to interact with 465 microRNAs) and 8 during differentiation (predicted to interact with 176 microRNAs). Together, these transcriptomic and regulatory differences appear to translate into distinct signaling strategies that support muscle growth. Specifically, NCT SCs exhibit greater activation of the mTOR/S6K pathway, which promotes protein synthesis and hypertrophic growth, consistent with enhanced muscle mass accretion in the modern commercial line (Xu et al., 2022b). In contrast, RBC2 SCs rely more heavily on Frizzled7-mediated Wnt/planar cell polarity signaling, which regulates cell polarity and tissue organization, suggesting a stronger dependence on developmental pathways that support muscle growth and remodeling (Xu et al., 2022a).

## Genetic selection impacts satellite cell heterogeneity

Since Schultz and Lipton (1982) first reported that SC proliferative capacity varies with age, extensive research has established that SCs represent a heterogeneous population. This heterogeneity is influenced by physiological status, developmental stage, muscle type, sex, and environmental conditions. Similar diversity has also been demonstrated in avian species. For example, McFarland et al. (1995) reported substantial variation among turkey SCs isolated from the pectoralis major muscle, despite its largely homogeneous Type IIb fiber composition (McFarland et al., 1995a). Based on proliferation behavior, they identified 73 distinct SC colonies with different growth rates. Fast-growing cells were more responsive to *fibroblast growth factor 2*, expressed higher levels of *fibroblast growth factor receptor 1* at the onset of proliferation, and produced greater amounts of heparan sulfate proteoglycan during differentiation (McFarland et al., 1995b). Follow-up studies further showed that fast-growing cells were more sensitive than slow-growing cells to the inhibitory effects of *transforming growth factor beta* on both proliferation and differentiation (Yun et al., 1997). Molecular profiling revealed over 5,300 genes differentially expressed between fast- and slow-growing cells during proliferation, with fast-growing cells enriched for gene programs involved in muscle development and structural maturation, whereas slow-growing cells upregulated genes associated with cell-cell communication, extracellular matrix interactions, ligand signaling, and cytokine activity (Yu et al., 2025). During differentiation, more than 1,400 genes were differentially expressed. Gene ontology analysis indicated that the fast-growing cells were enriched for genes involved in organ development and receptor signaling, while the slow-growing cells upregulated genes associated with cell proliferation and peptidase activator activity (Yimiletey et al., 2025). Together, these analyses indicate that the two SC populations differ not only in their proliferation rates and transcriptomic profiles, but also in their functional properties, further highlighting the biological significance of SC heterogeneity.

Genetic selection significantly alters the SC heterogeneity in broiler chickens. A comparative study using immunofluorescence staining to identify MYF-5, MYOD, and PAX7-expressing SCs revealed substantial differences between fast-growing Ross 708 (Aviagen Group) and slow-growing Red Ranger broilers. Muscles from the Ross strain exhibited a 32% larger population of triple-positive (MYF-5+:MYOD+:PAX7+) SC compared to the Red Ranger strain, indicating that intensive genetic selection for rapid growth has resulted in a more active SC pool (Tejeda et al., 2019). Consistently, a separate study in White Plymouth Rock chickens divergently selected for body weight reported a significantly higher proportion of MYF5 positive active SCs in the high weight line compared with the low weight line (Geiger et al., 2018). A recent single cell RNA sequencing study compared SC subpopulations from embryonic leg muscle between Daheng broilers, a cultivated breed, and Tibetan chickens, a native breed. Trajectory analysis revealed that SCs from Daheng broilers exhibited two bifurcation points in their developmental pathways that were absent in Tibetan chickens. This unique trajectory may contribute to their superior muscle growth and hypertrophic capacity, which appears to be established even before the onset of posthatch muscle hypertrophy (Li et al., 2025).

## Roles of satellite cell in wooden breast myopathy

Meat quality is influenced by the morphological features and cellular biology that govern skeletal muscle growth and development. With the production of heavier birds and increased breast muscle yield, the incidence of myopathies such as white striping (Kuttappan et al., 2013), spaghetti meat (Baldi et al., 2018), and wooden breast (WB) has risen (Sihvo et al., 2014). This review focuses specifically on WB, a fibrotic and necrotic myopathy, as the role of SCs in its pathogenesis has been more extensively investigated than in other breast muscle disorders.

Wooden breast syndrome was first reported in 2014, and it is now widely recognized that its incidence is positively associated with intensive genetic selection for heavier, fast-growing broilers (Sihvo et al., 2014; Petracci et al., 2019). WB-affected p. major muscles are characterized by discoloration, a turbid viscous coating on the outer surface, and a distinctly firm, hardened texture upon palpation (Sihvo et al., 2014; Bailey et al., 2015). Histologically, WB lesions feature myofiber degeneration and necrosis, inflammatory cell infiltration, increased collagen deposition, lipid accumulation, and progressive fibrosis (Sihvo et al., 2014; Velleman and Clark, 2015). Under normal conditions, SCs coordinate muscle repair through activation, proliferation, and differentiation to restore damaged myofibers; however, in WB this regenerative process is impaired, as evidenced by the accumulation of small-diameter fibers with sarcomere disorganization, implicating SC dysfunction as a driver of WB pathogenesis (Velleman et al., 2018; Velleman, 2023).

Previous transcriptomic (Marchesi et al., 2019; Xing et al., 2021), metabolomic (Abasht et al., 2016), and proteomic studies (Bottje et al., 2021) have demonstrated that hypoxia and oxidative stress (Abasht et al., 2016), together with dysregulated energy metabolism (Lake et al., 2019), mitochondrial dysfunction (Hosotani et al., 2020), and alterations in extracellular matrix (ECM) composition (Velleman, 2020; Pejskova et al., 2024), collectively contribute to the onset and progression of WB. More recent evidence has further implicated disrupted glucose metabolism (Bordini et al., 2024) as an integral component of the regulatory landscape underlying WB development. Several genes identified from these transcriptomic analyses have subsequently been functionally examined and shown to differentially regulate SC function in randombred chickens compared with commercial Ross 708 broilers, thereby providing a mechanistic link between whole-muscle molecular signatures and SC function (Velleman et al., 2021; 2022; 2024).

Recent advances in single-cell transcriptomics have begun to address this limitation. A single-cell RNA sequencing analysis comparing WB-affected and control p. major muscles identified 23 transcriptionally distinct cell clusters. This study reported an increased abundance of SCs in WB muscle and described a SC subpopulation that was largely unique to the WB condition. This WB-associated SC subcluster was characterized by elevated expression of *APOA1*, *COL3A1*, and *COL1A1*, with enrichment of pathways related to actin cytoskeleton organization, ECM receptor interaction, and focal adhesion, and followed a distinct differentiation trajectory. In addition, the analysis identified a macrophage cluster that was almost exclusive to WB muscle, marked by expression of *CCL4*, *CRISPLD*, *CA9*, *IL5RA*, *BATF3*, and *SLC16A3*. This macrophage population exhibited active communication with SCs under normal conditions, suggesting that disrupted immune-SC interactions may play an important role in WB pathogenesis (Zhao et al., 2024). Notably, a separate study using Visium spatial transcriptomics to compare p. major muscle from WB-affected and control birds suggested that WB-affected muscle undergoes reprogramming of lipid metabolism, characterized by upregulation of lipid transporters and genes in the peroxisome proliferator-activated receptor pathway in lipid-laden macrophages. In addition, the pathways associated with connective tissue were primarily related to extracellular matrix and collagen organization, suggesting early tissue remodeling or the initiation of fibrosis in WB tissue. All of these changes may directly impair normal SC function (Wang et al., 2024). An integrative analysis of single-cell and spatial transcriptomics will be needed to better understand how the cellular microenvironment shapes SC heterogeneity and influences their function during WB progression.

To investigate a potential cell-autonomous role of SCs in the pathogenesis of WB myopathy, a separate study examined SCs isolated from WB-affected and normal p. major muscles (Pejskova et al., 2024). SCs derived from WB muscle exhibited impaired proliferation, with most cells failing to reach more than 50% confluency before growth arrest. This proliferative defect was independent of p38 and ERK1/2 signaling. Although SC proliferation was impaired, WB did not appear to alter the overall differentiation capacity of these cells. Transcriptomic analysis of SCs from WB-affected chickens showed downregulation of gene sets related to heparan sulfate glycosaminoglycan biosynthesis, extracellular matrix–receptor interaction, and focal adhesion. Given the established involvement of syndecans in these processes, syndecan-4 (SDC4) shedding was investigated further. WB myopathy was associated with a tendency toward reduced SDC4 expression and increased ectodomain shedding. Furthermore, SDC4 overexpression was linked to reduced SC proliferation and altered myogenesis, while blocking peptides derived from the SDC4 ectodomain also altered SC proliferation. Together, these findings support a role for SDC4 in regulating SC function in WB. Although rodent knockout models have implicated SDC4 in SC activation (Cornelison et al., 2004), the present study more directly supports its involvement in SC proliferation and differentiation rather than SC activation itself during WB pathogenesis (Pejskova et al., 2024).

## Conclusions and future perspectives

In conclusion, intensive genetic selection for rapid growth in meat-type poultry has led to pronounced myofiber hypertrophy, driven in large part by SC-dependent protein accretion during the post-hatch period. These advances have substantially improved breast muscle yield and overall production efficiency, while also shaping SC behavior in ways that appear to differ between broilers and turkeys, despite broadly similar selection strategies. Notably, fibrotic and necrotic breast muscle disorders such as WB have been reported in broiler chickens but not in turkeys, highlighting species-specific differences in SC regulation and muscle adaptation. As discussed throughout this review, a deeper understanding of SC biology will be essential for supporting continued improvements in muscle growth while minimizing the risk of muscle myopathies. Moreover, recent advances in single-cell RNA sequencing and spatial transcriptomic platforms, including Xenium and MERFISH, offer powerful tools to dissect SC heterogeneity at single-cell resolution and to define how local cellular neighborhoods regulate SC behavior. Given that SCs undergo asymmetric division, in which one daughter cell preserves the stem cell pool while the other commits to myogenic differentiation (Kuang et al., 2007), it will be especially important to determine whether genetic selection has altered the composition of SC subpopulations and how the niche shapes this diversity. To date, most single-cell RNA sequencing studies in poultry breast muscle have primarily characterized transcriptomic changes during development (Li et al., 2020; Zhang et al., 2026), rather than examining how genetic selection alters cellular composition and transcriptomics, particularly in SCs. Looking forward, an important area of future research will be to determine how genetic selection and nutritional paradigms reshape the SC pool and its microenvironment. Such knowledge could ultimately be applied to develop poultry lines that are more resilient to environmental stressors and disease challenges, with important implications for both animal well-being and poultry production efficiency.
